# The segetal flora of Italy: an occurrence dataset from relevés in winter cereals and allied crop types

**DOI:** 10.3897/phytokeys.161.53915

**Published:** 2020-10-02

**Authors:** Emanuele Fanfarillo, Marta Latini, Mauro Iberite, Giovanna Abbate

**Affiliations:** 1 Department of Environmental Biology, Sapienza University of Rome, P.le A. Moro 5, 00185 Rome, Italy Sapienza University of Rome Rome Italy

**Keywords:** weed, agriculture, agroecosystem, arable flora, archaeophyte, observation, wheat, winter annual crop

## Abstract

The segetal flora of winter crops includes mostly native or archaeophyte annual species that are often strong specialists of their habitats. Threatened by the intensification of agriculture, segetal flora is particularly valuable from a perspective of biodiversity conservation and evolution. Moreover, it contributes to maintain biodiversity in agroecosystems and provides several ecosystem services. The dataset here described was set up to provide the first inventory of the segetal flora of Italian winter cereal crops and allied crop types, the latter including flax and autumn-sown legumes. It includes 24,676 georeferenced occurrence data deriving from 1,240 floristic and phytosociological relevés. The data were collected from the greater part of Italian territory, in a temporal range spanning from 1946 to 2018.

## Introduction

The concept of “weed” is very subjective, as any plant that interferes with human activities can be considered as such, implying the existence of agricultural weeds, environmental weeds, ruderal weeds and many others. Weeds of arable land are almost exclusively annual and are called “agrestals” or “segetals” (Holzner 1982). For decades, they have been negatively affected by the intensification of agriculture all over Europe (Storkey et al. 2012; Richner et al. 2015; Janssen et al. 2016; Pannacci et al. 2017; Woźniak 2020). In recent years, many studies highlighted the ecological and agronomic benefits of these species in agricultural systems, including the provision of ecosystem services, such as support to biodiversity, storage of crop genetic resources, pest regulation and soil protection (Hammer et al. 1997; Marshall et al. 2003; Storkey and Neve 2018). At middle and high latitudes, segetal plants can be divided into two main groups according to their phenology, the latter depending on the crops they colonise: species of winter crops, like wheat and species of summer crops, like maize. In Europe, winter-annual crops host mostly native or archaeophyte segetal species, which are often strong specialists of these habitats (Lososová et al. 2004; Brullo et al. 2007; Brullo and Guarino 2007; Abbate et al. 2013; Nowak et al. 2015; Latini et al. 2020). Several anecophytes are present amongst them: “homeless weeds” without a natural habitat, which recently evolved under the pressure of agriculture and developed biological and ecological features similar to those of crop species (Zohary 1962; McElroy 2014). For all these reasons, the segetal flora of winter cereal crops owns a peculiar value from the perspectives of biodiversity conservation and evolution.

The here presented dataset is available in GBIF (Fanfarillo et al. 2020a) and includes 24,676 records. Of the latter, 2,878 were newly acquired through field sampling and 21,798 were retrieved from literature. The dataset was set up to define the first inventory of the segetal flora of Italian winter cereal crops and allied crop types (from here on simply “segetal flora”), i.e. flax and autumn-sown legumes (Fanfarillo et al. 2020b). It is the first contribution by the Laboratory of Systematic Botany and Floristics, Department of Environmental Biology, Sapienza University of Rome to the GBIF, which approved it as a data editor in March 2020 (responsible person: Mauro Iberite; technical contact: Marta Latini).

In the light of what is stated above, the main aims of the present paper are the description and presentation of this recently-released dataset, providing information on its usefulness and possible future applications.

## Related project

### Project title

Plant biodiversity in traditional agroecosystems of Italy: a floristic and ecological multi-scale analysis based on geodatabases.

### Identifier

RM118164361D0EE4 (Progetto di Ricerca Medio, Sapienza University of Rome).

### Personnel

Giovanna Abbate, Mauro Iberite, Marta Latini, Emanuele Fanfarillo

### Funding

Sapienza University of Rome, Piazzale Aldo Moro 5, 00185, Rome, Italy.

### Project data

The disappearance of traditional agroecosystems and the consequent biodiversity loss due to changes in agriculture are receiving increasing attention in Europe. The use of databases on plant taxonomical, distributional, ecological and functional traits is of crucial importance in conservation actions. The need to improve monitoring and reporting activities by improving the quality of biodiversity data is also underlined by the European Biodiversity Strategy. This project aimed at fulfilling a global analysis of the plant diversity existing in the traditional agroecosystems of Italy, knowledge of which is currently lacking, by means of the collection, digitisation and processing of original and archival data. The proposed actions concerned: the preparation of thematic databases on segetal flora and vegetation, including the features of plant species and communities; the analysis of data at different spatial and temporal scales; the production of thematic maps on plant diversity and its related topics; the development of new methods to estimate the nature value of agroecosystems; the detection of bio-indicator plant species for floristic richness, agricultural intensity and environmental quality. Special attention was given to winter arable plants and communities, currently at high risk of disappearance in Europe. The achieved results provided an important basis for any future research, with special regards to the definition of conservation strategies for plant diversity in European rural areas.

## Methods

The occurrence data were retrieved through extensive literature searches and intensive field samplings, the latter being carried out in the greater part of Italy to fill the knowledge gaps in some geographic areas. Literature data were selected using a habitat-based criterion: only the records for taxa unambiguously reported to grow in winter cereals, flax and autumn-sown legumes were collected. Consequently, all the records with no or with generic information on the growing habitat (e.g. “fields” or “cultivated land”) were excluded. Likewise, records of taxa identified to the genus or higher level, doubtful identifications, nomenclatural ambiguities and crop species were not considered. The bibliographic source of each record is available upon request to the authors.

All the occurrence data were georeferenced. Geographic coordinates (decimal latitude and decimal longitude), geodetic datum and a value of uncertainty for coordinates were attributed to each single record. The geographic coordinates were manually attributed, based on the descriptions of the relevé location provided in the original source. If coordinates were already available, they were converted in WGS84 geodetic datum, when differently expressed. The uncertainty of geographic positions was estimated according to the 9-degree scale defined by Murphey et al. (2004) and then converted into metres, as requested by GBIF (1, 100, 500, 1000, 5000, 10,000, 50,000 m or accordingly higher, if only the administrative region/country were given for data, following the same method used in Küzmič et al. 2020). Georeferencing historical data was often challenging due to vague information on the collection place or to the report of non-localisable toponyms. In these cases, the records where georeferenced as accurately as possible on a wider scale (e.g. the “comune” when the reported locality within the “comune” could not be identified).

The taxonomic nomenclature was updated according to the latest standards available for the Italian flora (Bartolucci et al. 2018a, b, c; Galasso et al. 2019; Bartolucci et al. 2019 for native species; Galasso et al. 2019 for alien species).

The dataset was validated using GBIF Data Validator (https://www.gbif.org/tools/data-validator) and was published using GBIF Integrated Publishing Toolkit (IPT) publishing platform (https://cloud.gbif.org/eca). Once the data were imported in GBIF, the nomenclature was automatically referred to the GBIF Backbone Taxonomy (GBIF Secretariat 2019). Nevertheless, original names are available for consultation for each record.

## Results

### Dataset description

**Object name**: Darwin Core Archive Segetal flora of Italy

**Character encoding**: UTF-8

**Format name**: Darwin Core Archive format

**Format version**: 1.0

**Distribution**: https://cloud.gbif.org/eca/archive.do?r=segflorit

**Publication date of data**: 09-07-2020

**Language**: English

**Licences of use**: Creative Commons Attribution (CC-BY) 4.0 License

**Metadata language**: English

**Date of metadata creation**: 09-04-2020

**Hierarchy level**: Dataset

**The fields provided by the “Segetal flora of Italy” dataset are**:

occurrenceID, basisOfRecord, eventDate, scientificName, kingdom, taxonRank, decimalLatitude, decimalLongitude, geodeticDatum, coordinateUncertaintyInMetres, continent, country, countryCode, stateProvince, organismQuantity, organismQuantityType.

### Taxonomic coverage

Most of the records belong to the class Magnoliopsida (20,307 records; 82% of the total), followed by Liliopsida (4,208 records; 17%) and Polypodiopsida (117 records; 0.5%). Though, on the basis of the most recent results summarised by the APG (Stevens 2001 onwards), this classification is outdated, the technical schemes of the GBIF Backbone Taxonomy impose following this taxonomic scheme (GBIF Secretariat 2019).

Within Magnoliopsida, the most represented orders are Asterales (15%), Ranunculales (12%), Caryophyllales (11%) and Fabales (8%). Poales (14%) is the most represented order within Liliopsida. The records in the dataset belong to 53 families, 340 genera and 859 taxa. The five most represented families, genera and species are shown in Tables 1–3, respectively.

**Table 1.** The five most represented families in the “Segetal flora of Italy” dataset: number of records, percentage of records, number of genera and number of species are reported for each family.

Family	No. records	% of records	No. genera	No. species
Poaceae	3,468	14.1	46	100
Asteraceae	3,100	12.6	69	126
Fabaceae	1,925	7.8	19	95
Apiaceae	1,658	6.7	30	43
Papaveraceae	1,582	6.4	3	12

**Table 2.** The five most represented genera in the “Segetal flora of Italy” dataset: family, number of records, percentage of records and number of species are reported for each genus.

**Genus (Family)**	**No. records**	% **of records**	**No. species**
*Papaver* (Papaveraceae)	1,243	5.0	5
*Veronica* (Plantaginaceae)	690	2.8	12
*Lysimachia* (Primulaceae)	689	2.8	3
*Ranunculus* (Ranunculaceae)	656	2.7	11
*Lolium* (Poaceae)	641	2.6	4

**Table 3.** The five most represented species in the “Segetal flora of Italy” dataset: family, number of records and percentage of records are reported for each species.

**Species (Family)**	**No. records**	% **of records**
*Papaver rhoeas* L. (Papaveraceae)	917	3.7
*Convolvulus arvensis* L. (Convolvulaceae)	564	2.3
*Ranunculus arvensis* L. (Ranunculaceae)	496	2.0
*Lysimachia arvensis* (L.) U.Manns & Anderb. (Primulaceae)	466	1.9
*Polygonum aviculare* L. (Polygonaceae)	429	1.7

### Taxonomic ranks

**Kingdom**: Plantae

**Phylum**: Tracheophyta

**Class**: Magnoliopsida, Liliopsida, Polypodiopsida.

**Order**: Alismatales, Apiales, Asparagales, Asterales, Boraginales, Brassicales, Caryophyllales, Cucurbitales, Dipsacales, Equisetales, Ericales, Fabales, Gentianales, Geraniales, Lamiales, Liliales, Malpighiales, Malvales, Myrtales, Oxalidales, Piperales, Poales, Polypodiales, Ranunculales, Rosales, Salviniales, Santalales, Saxifragales, Solanales.

**Family**: Amaranthaceae, Amaryllidaceae, Apiaceae, Araceae, Aristolochiaceae, Asparagaceae, Asteraceae, Boraginaceae, Brassicaceae, Campanulaceae, Caprifoliaceae, Caryophyllaceae, Convolvulaceae, Cucurbitaceae, Cyperaceae, Dennstaedtiaceae, Elatinaceae, Equisetaceae, Euphorbiaceae, Fabaceae, Gentianaceae, Geraniaceae, Heliotropiaceae, Hypericaceae, Iridaceae, Juncaceae, Lamiaceae, Liliaceae, Linaceae, Lythraceae, Malvaceae, Marsileaceae, Onagraceae, Orobanchaceae, Oxalidaceae, Papaveraceae, Plantaginaceae, Poaceae, Polygonaceae, Portulacaceae, Primulaceae, Ranunculaceae, Resedaceae, Rosaceae, Rubiaceae, Santalaceae, Saxifragaceae, Scrophulariaceae, Solanaceae, Thymelaeaceae, Urticaceae, Verbenaceae, Violaceae.

### Spatial coverage

**Figure 1.**
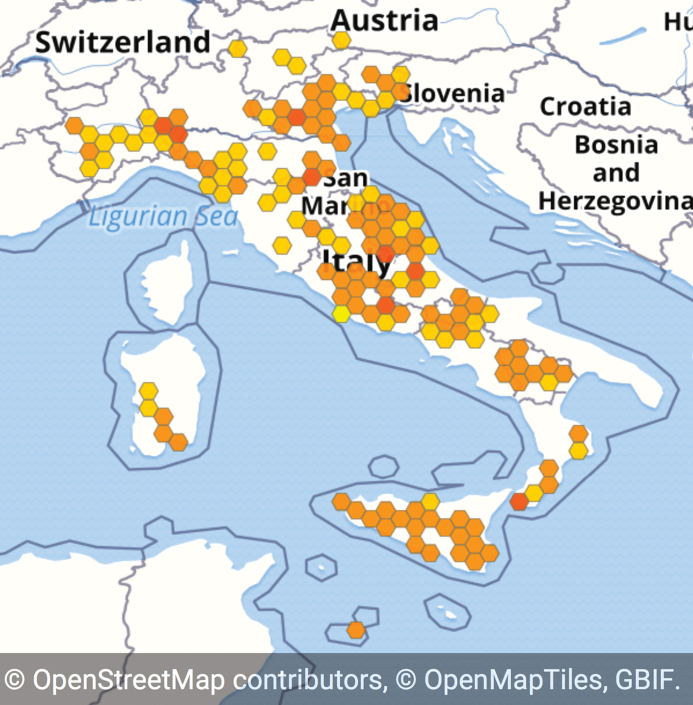
Geographical coverage of the “Segetal flora of Italy” dataset. Different colours express the different number of records per cell (darker colours = higher number of records).

The species occurrences stored in the dataset were recorded from the greater part of the Italian territory (Fig. 1 – Bounding box coordinates: 35°23'60"N, 6°30'0"E; 47°12'0"N, 18°36'0"E). Regarding administrative regions, the highest numbers of records are from Sicily, Veneto, Lombardy and Latium (Table 4). So far, no data could be retrieved for Valle d’Aosta and Apulia. A total of 17.3% of the coordinates had an uncertainty of 1 m; 33.2% of 1,000 m; 5.9% of 5,000 m; 25.2% of 10,000 m; 5.6% of 50,000 m; 12.7% of 100,000 m.

**Table 4.** Number and percentage of records per administrative region in the “Segetal flora of Italy” dataset.

**Administrative region**	**No. records**	% **on the total**
Sicily	6035	24.5
Veneto	2680	10.9
Lombardy	2480	10.1
Latium	2086	8.4
Emilia-Romagna	1797	7.3
Abruzzo	1785	7.2
Marche	1725	7.0
Umbria	1652	6.7
Calabria	1120	4.5
Basilicata	692	2.8
Molise	648	2.6
Friuli Venezia Giulia	559	2.3
Tuscany	396	1.6
Piedmont	373	1.5
Sardinia	298	1.2
Campania	249	1.0
Trentino-Alto Adige	74	0.3
Liguria	27	0.1

### Temporal coverage

**Figure 2.**
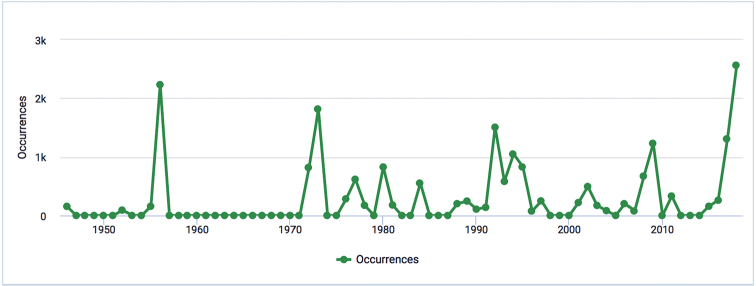
Temporal coverage of the “Segetal flora of Italy” dataset (occurrences per year).

The dataset includes species occurrences recorded from 1946 to 2018 (Fig. 2). Most of the records were collected in the 1970s, 1990s and 2010s. The date of collection is available for 84% of the data (20,703 records).

**Figure 3.**
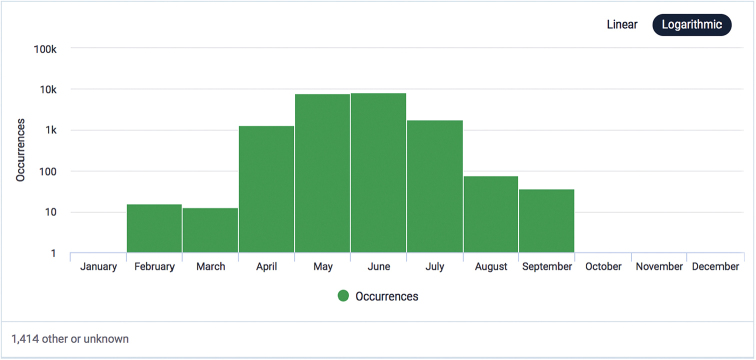
Temporal coverage of the “Segetal flora of Italy” dataset (occurrences per month).

As expected, a high seasonality characterises the dataset. Most of the occurrences were recorded in spring and early summer. The months of greatest occurrence of the investigated taxa are, respectively, June, May, July and April (Fig. 3).

### Interest and use of the dataset

The “Segetal flora of Italy” dataset was the basis for the definition of the first inventory of the segetal flora of Italian winter cereal crops and allied crop types (Fanfarillo et al. 2020b). The latter is one of the first of its kind for European countries, following the French one (Aboucaya et al. 2000; Cambecédes et al. 2012). Part of the stored data was used to highlight the influence of the geo-environmental factors and the patterns of co-occurrence of rare and threatened arable species in winter arable plant communities of mainland Italy (Fanfarillo et al. 2020c, d). Moreover, another subset of the data contributed, in the form of vegetation plots, to the establishment of the European Weed Vegetation Database (Küzmič et al. 2020). Besides GBIF, the occurrences stored in the ”Segetal flora of Italy” database will be also stored in other important biodiversity data repositories, such as the Italian Wikiplantbase #Italia (Peruzzi et al. 2019 onwards; Dipartimento di Biologia, Università di Pisa 2020).
